# Multicenter Evaluation of the BD Phoenix CPO Detect Test for Detection and Classification of Carbapenemase-Producing Organisms in Clinical Isolates

**DOI:** 10.1128/JCM.01752-19

**Published:** 2020-04-23

**Authors:** Vicki Whitley, Susan Kircher, Tracey Gill, Janet A. Hindler, Susan O’Rourke, Charles Cooper, Anagha Tulpule, Gerald A. Denys

**Affiliations:** a Becton, Dickinson and Company, BD Life Sciences-Integrated Diagnostic Solutions, Sparks, Maryland, USA; b David Geffen School of Medicine, University of California Los Angeles, Los Angeles, California, USA; c Indiana University School of Medicine, Indianapolis, Indiana, USA; Medical College of Wisconsin

**Keywords:** Ambler class carbapenemase, carbapenem resistance, carbapenemase-producing organisms, carbapenemase-resistant *Enterobacteriales*, Phoenix CPO detect

## Abstract

Limited treatment options contribute to high morbidity/mortality rates with carbapenem-resistant, Gram-negative bacterial infections. New approaches for carbapenemase-producing organism (CPO) detection may help inform clinician decision-making on patient treatment and infection control. BD Phoenix CPO detect (CPO detect) detects and classifies carbapenemases in *Enterobacterales*, Acinetobacter baumannii, and Pseudomonas aeruginosa during susceptibility testing. The clinical performance of CPO detect is reported here. *Enterobacterales*, Acinetobacter baumannii, and Pseudomonas aeruginosa isolates were evaluated across three sites using CPO detect and a composite reference method (RM); the latter was comprised of the modified carbapenem inactivation method and a MIC screen for ertapenem, imipenem, and meropenem. Multiplex PCR testing was also utilized for Ambler class determination. Positive and negative percentages of agreement (PPA and NPA, respectively) between CPO detect and the RM were determined. The PPA and NPA for *Enterobacterale*s were 98.5% (confidence intervals, 96.6%, 99.4%) and 97.2% (95.8%, 98.2%), respectively. The A. baumannii PPA and NPA, respectively, were 97.1% (90.2%, 99.2%) and 97.1% (89.9%, 99.2%). The P. aeruginosa PPA and NPA, respectively, were 95.9% (88.6%, 98.6%) and 92.3% (86.7%, 95.6%). The PPA values for carbapenemase class designations for all organisms combined and *Enterobacterale*s alone, respectively, were 95.3% (90.2%, 97.8%) and 94.6% (88.8%, 97.5%) for class A, 94.0% (88.7%, 96.6%) and 96.4% (90.0%, 98.8%) for class B, and 95.0% (90.1%, 97.6%) and 99.0% (94.4%, 99.8%) for class D carbapenemases. NPA values for all organisms and *Enterobacterales* alone ranged from 98.5% to 100%. CPO detect provided accurate detection and classification of CPOs for the majority of isolates of *Enterobacterales*, Acinetobacter baumannii, and Pseudomonas aeruginosa tested.

## Abstract (precis)

Limited treatment options contribute to high morbidity/mortality rates with carbapenem-resistant, Gram-negative bacterial infections. New approaches for carbapenemase-producing organism (CPO) detection may help inform clinician decision-making on patient treatment and infection control. BD Phoenix CPO detect (CPO detect) detects and classifies carbapenemases in *Enterobacterales*, Acinetobacter baumannii, and Pseudomonas aeruginosa during susceptibility testing.

## INTRODUCTION

Approximately 2.8 million people are infected with antibiotic-resistant bacteria each year in the United States; at least 35,000 die as a result of antibiotic resistance (Centers for Disease Control and Prevention, National Center for Emerging and Zoonotic Infectious Diseases [NCEZID], Division of Healthcare Quality Promotion [DHQP]; https://www.cdc.gov/ncezid/dhqp/index.html, accessed 7 January 2019). A large number of these mortalities are caused by multidrug-resistant (MDR), Gram-negative organisms (1, 2), which continue to represent a major health concern, especially in hospital settings (3–5), where mortality rates due to MDR range from 30 to 70% (6, 7). Recent data suggest that anywhere from 5 to 60% of infections involve antibiotic-resistant organisms (8, 9). Carbapenems are among a diminishing list of effective antibiotic classes for the treatment of MDR infections due to Gram-negative bacteria (10, 11). Their efficacy, however, continues to wane with the increased proliferation of carbapenem-resistant organisms, including *Enterobacterales*, Acinetobacter baumannii, and Pseudomonas aeruginosa (12).

Carbapenem resistance in Gram-negative bacteria can be mediated by at least two mechanisms. One involves expression of cephalosporinases (such as AmpC β-lactamase) or extended-spectrum β-lactamases (ESBLs), with a simultaneous accumulation of mutations in genes that code for outer membrane porins, which decrease cell wall permeability to carbapenem antibiotics or increased efflux pump activity (13). Alternatively, Gram-negative bacteria (including *Enterobacterales*, A. baumannii, and P. aeruginosa) can produce carbapenemase enzymes which hydrolyze carbapenems (12). These organisms are often referred to as carbapenemase-producing organisms (CPOs). Carbapenemases are members of the Ambler molecular class A, B, and D β-lactamase enzymes (14).

Current recommendations from both the Clinical and Laboratory Standards Institute (CLSI) and the European Committee on Antimicrobial Susceptibility Testing (EUCAST) state that carbapenemase testing may be important for epidemiology and infection control purposes to inform containment strategies (e.g., in hospitals or during outbreaks). Carbapenem susceptibility test results of a MIC or disk diffusion method should guide antimicrobial selection for treatment purposes (15, 16). While both phenotypic and genotypic tests are utilized to determine the presence of CPOs, each has advantages and limitations. Genotypic tests (e.g., nucleic acid amplification tests) can detect the presence of different genes and have a relatively fast turnaround time; however, they are costly and are generally targeted toward genes coding for the most common carbapenemases. Phenotypic assays, which often require more hands-on time, have the advantage of being able to detect previously unidentified carbapenemases (17, 18), although they may fail to detect some CPOs that express carbapenemase enzymes at low levels (19, 20).

BD Phoenix CPO detect (CPO detect; Becton, Dickinson and Company; BD Life Sciences-Integrated Diagnostic Solutions, Sparks, MD, USA) is a qualitative, confirmatory, growth-based phenotypic test that detects carbapenemase production in isolates of *Enterobacterales*, P. aeruginosa, and A. baumannii as part of routine antimicrobial susceptibility testing (AST) when the Phoenix Automated Microbiology System is used. CPO detect utilizes meropenem, doripenem, temocillin, and cloxacillin, either alone or in combination with various chelators and β-lactamase inhibitors, to detect and categorize carbapenemases and is available in two panel configurations (with 2 and 9 wells). The 2-well format detects carbapenemase presence or absence; in addition to detecting the presence of a carbapenemase, the 9-well format reports the Ambler classification (A, B, and D) for carbapenemases produced by isolates of *Enterobacterales*, A. baumannii, and P. aeruginosa. Here, the accuracy of CPO detect was assessed by comparing it to a composite reference method (RM) using clinical isolates and to previously obtained results from challenge isolates of *Enterobacterales*, A. baumannii, and P. aeruginosa.

## MATERIALS AND METHODS

Our study was a multicenter, prospective evaluation of CPO detect compared to the RM using clinical and challenge isolates representing species included in the Phoenix ID/AST taxon list for *Enterobacterales*, P. aeruginosa, and A. baumannii or A. baumannii complex. Clinical isolates (fresh and stock) were obtained from adult and pediatric patients seen at UCLA Healthcare, Los Angeles, CA (UCLA) and Indiana University Health, Indianapolis, IN (IUHP). Additional clinical stock isolates were tested at the R&D Laboratory at BD Life Sciences-Integrated Diagnostic Solutions. Challenge isolates were evenly divided and tested at all three sites. Institutional review board approval was obtained for this study, as appropriate; only deidentified remnant bacterial isolates were used in this study. This article was prepared according to Standards for Reporting Diagnostic Accuracy (STARD) guidelines (21).

### Clinical and challenge isolate sets.

Challenge isolates were obtained from several domestic and international sources, including the Centers for Disease Control and Prevention (CDC; FDA AR Isolate Bank) isolates. Genotypic characterizations of carbapenemases were previously obtained for these isolates by using various molecular techniques, including multiplex PCR and sequencing of extracted β-lactamases. At each of the three test sites, challenge isolates were tested for carbapenemase production by using the modified carbapenem inactivation method (mCIM), and carbapenem MIC testing was performed using the Phoenix system to ensure that the organisms had not lost carbapenem resistance markers.

All sites tested isolates obtained from clinical specimens. Fresh clinical isolates were collected and stored on agar plates for 7 days or less and were never frozen. Stock isolates were obtained from clinical specimens and stored on agar plates for more than 7, but less than 60, days or stored frozen for less than 3 years, when possible. Additional stock isolates were distributed from BD Life Sciences-Integrated Diagnostic Solutions to the external sites to ensure that each site would test a minimum of 10 isolates from each Ambler class.

### Isolate inclusion and Phoenix panel preparation.

Fresh isolates were subcultured once and frozen isolates subcultured twice on Trypticase soy agar with 5% sheep blood (TSA II with 5% SB) (Becton, Dickinson and Company, BD Life Sciences-Integrated Diagnostic Solutions) and incubated at 35°C ± 2°C for 18 to 24 h in ambient air prior to testing. A single Phoenix panel, containing CPO detect in the 9-well configuration, MIC tests for ertapenem, imipenem, and meropenem, and identification tests, was inoculated with each test isolate according to the Phoenix System manufacturer's instructions and study protocol (Becton, Dickinson and Company, BD Life Sciences-Integrated Diagnostic Solutions). The Phoenix system utilized software of version 6.21A/5.95C or higher (August 2016). Following Phoenix panel inoculation, a purity plate was prepared and incubated at 35°C ± 2° for 18 to 24 h in ambient air.

**FIG 1:**
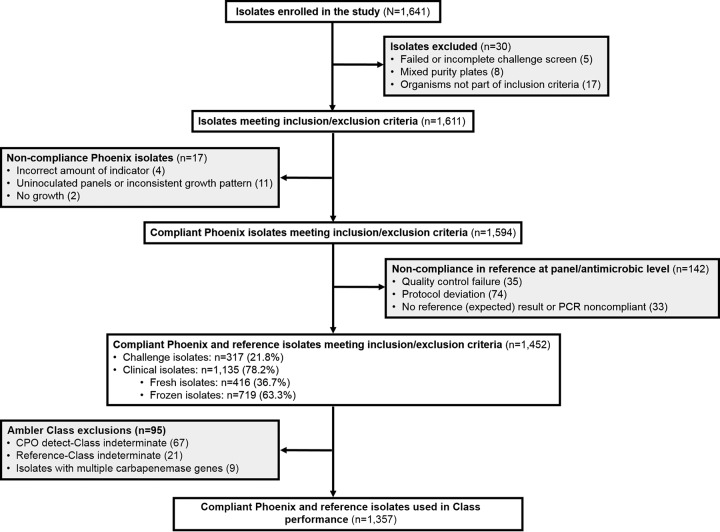
Reconciliation for enrolled isolates utilized during the Phoenix CPO detect study. Inclusion criteria: (1) isolates obtained from adult and pediatric populations; (2) organisms included in the Phoenix ID/AST taxa list for *Enterobacterales*, P. aeruginosa, and A. baumannii or A. baumannii complex; (3) one isolate of a species from a single patient. No specimens were collected solely for the purpose of this study.

A total of 1,641 Gram-negative clinical and challenge isolates were enrolled in this study; Fig. 1 details isolate reconciliation. Results from a total of 1,452 isolates (clinical and challenge) were compliant by both CPO detect and the RM and evaluated. Of these, 1,135 (78.2%) were clinical and 317 (21.8%) were challenge isolates. *Enterobacterales* represented 75.7% of the isolates tested; A. baumannii and P. aeruginosa represented 9.5% and 14.8% of test isolates, respectively (Table 1). All sites tested both clinical and challenge isolates. UCLA, IUHP, and BD Life Sciences-Integrated Diagnostic Solutions tested 344, 358, and 750 isolates, respectively.

**TABLE 1:** CPO detect test performance for carbapenemase detection in all isolates^*a*^

Organism or group	Total no. of tests	No. of results that were:						No. of tests/total, % (95% CI)	
		RM pos	TP	FN	RM neg	TN	FP	PPA	NPA
*Enterobacterales*^*b*^	1,099	343	338	5	756	735	21	338/343, 98.5 (96.6, 99.4)	735/756, 97.2 (95.8, 98.2)
Acinetobacter baumannii	138	70	68	2	68	66	2	68/70, 97.1 (90.2, 99.2)	66/68, 97.1 (89.9, 99.2)
Pseudomonas aeruginosa	215	73	70	3	142	131	11	70/73, 95.9 (88.6, 98.6)	131/142, 92.3 (86.7, 95.6)
All organisms	1,452	486	476	10	966	932	34	476/486, 97.9 (96.3, 98.9)	932/966, 96.5 (95.1, 97.4)

a Abbreviations: CPO, carbapenemase-producing organism; RM, reference method; Pos, positive; TP, true positive; FN, false negative; Neg, negative; TN, true negative; FP, false positive; PPA, positive percent agreement; NPA; negative percent agreement; CI, confidence interval.

b The NPA carbapenemase detection performance for *Enterobacterales* challenge isolates (*n* = 210) was 77.2% (95% CI, 64.2%, 87.3%). Thirteen false positives were observed from 57 isolates that were negative for carbapenemase production by the reference method. These isolates included the following organisms: 8 K. pneumoniae species isolates, 1 Providencia stuartii isolate, 1 Proteus mirabilis isolate, 1 Klebsiella oxytoca isolate, 1 E. coli isolate, and 1 Klebsiella aerogenes isolate.

### RM.

**FIG 2:**
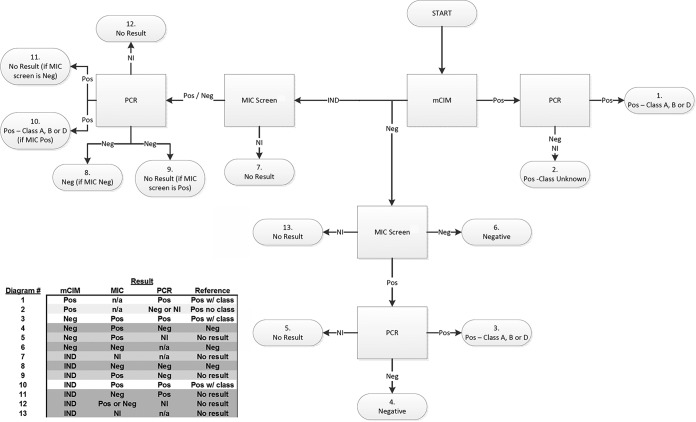
Decision flowchart for the composite reference method. Isolate testing was performed at each site using MIC screening and mCIM methods during the trial. Multiplex-PCR testing was performed by BD Life Sciences-Integrated Diagnostic Solutions, as needed, according to the decision flowchart for the composite reference method. Abbreviations: Neg, negative; Pos, positive; IND, indeterminate; NI, not interpretable; n/a, not applicable; w/, with.

The RM for clinical isolates followed a defined algorithm (Fig. 2) that was agreed to by the FDA prior to the study. Phenotypic results were obtained using mCIM for *Enterobacterales*, P. aeruginosa, and A. baumannii (22). MIC screening results were obtained with the Phoenix panel. The MIC screen used predetermined threshold values based on EUCAST and CLSI guidelines (15, 16). The RM MIC results obtained by the carbapenem agents present in the Phoenix panel were completely separate and independent from the CPO detect test and were used in the predefined algorithm to establish the need for multiplex PCR of any isolate.

Multiplex PCR is the genotypic method that was used to confirm the presence of specific carbapenemase-encoding genes. The frequency of PCR testing followed the reference flow chart in Fig. 2. Two laboratory-developed multiplex PCR assays were utilized to amplify eight of the most common carbapenemase-encoding genes in Ambler classes A, B, and D. One of the laboratory-developed PCR assays (multiplex PCR assay 1) was used for *Enterobacterales*, P. aeruginosa, and A. baumannii. This assay amplified *bla*_KPC_, *bla*_NDM_, *bla*_IMP_, *bla*_VIM_, and *bla*_OXA-48_-like genes (23). The second multiplex assay (multiplex PCR assay 2) was used only for P. aeruginosa and A. baumannii (24) and amplified three additional commonly encountered OXA-type genes (*bla*_OXA-23_, *bla*_OXA-24_, and *bla*_OXA-58_). Both methods were adapted from those referenced in other publications (24–26) and were optimized to ensure that each of the primer sets generated the correct amplicon. Table S1 lists the gene variants identified by PCR in this study.

In addition, RM MIC testing (as listed above for the RM algorithm determination) results were calculated for isolates that were CPO positive by the RM and susceptible (not including intermediate MIC results) to one or more carbapenems, according to CLSI interpretive criteria (16) (MICS for *Enterobacterales*, ≤1 μg/ml for meropenem, ≤1 μg/ml for imipenem, and ≤0.5 μg/ml for ertapenem; MICs for A. baumannii and P. aeruginosa, ≤2 μg/ml for imipenem and meropenem). Susceptible isolates included 32/343 (9.3%) *Enterobacterales*, 0/70 (0%) A. baumannii, and 0/73 (0%) P. aeruginosa isolates and were susceptible to at least one of the tested carbapenems (data not shown).

### Data and analysis.

CPO detect results for carbapenemase production were compared to RM results obtained during the study for clinical isolates and compared to RM results previously established for challenge isolates. The overall positive percent agreement (PPA) and negative percent agreement (NPA) were calculated, and the numbers of false-negative and false-positive results were determined for all organisms grouped together and also stratified by organism or organism group (*Enterobacterales*). Similarly, CPO results for Ambler classification were compared to RM results. The PPA and NPA were calculated together with 95% confidence intervals (CI) using standard statistical methods (21).

## RESULTS

### Carbapenemase detection.

Among all isolates evaluated in this study, 486/1,452 (33.5%) were CPOs by the RM. As shown in Table 1, PPA and NPA values for CPO detect for all organisms were 97.9% (95% CI, 96.3%, 98.9%) and 96.5% (95% CI, 95.1%, 97.4%), respectively. The PPA and NPA values were similar across organism types and ranged from 95.9% to 98.5% and from 92.3% to 97.2%, respectively. For *Enterobacterales* alone, 31.2% were identified by the RM as CPOs, with CPO detect resulting in values that were falsely positive 1.9% and falsely negative 0.5% of the time. For the nonfermenting organisms, 50.7% of A. baumannii and 34.0% of P. aeruginosa isolates were identified by the RM as CPOs. False-positive and false-negative values of 1.4% and 1.4%, respectively, were observed for A. baumannii, and false-positive and false-negative values of 5.1% and 1.4%, respectively, were observed for P. aeruginosa. A further breakout of CPO detect performance by organism is shown in Table S2 in the supplemental material.

### Carbapenemase classification.

CPO detect provided an Ambler classification for 394/486 (81.1%) isolates that were CPO positive by the RM. Sixty-four of 486 (13.2%) of the isolates were Ambler class A, B, or D or indeterminate by the RM but were indeterminate by CPO detect (Table 2). CPO detect correctly classified 122 of 128 Ambler class A (PPA of 95.3% [95% CI, 90.2%, 97.8%]), 126 of 134 class B (PPA of 94.0% [95% CI, 88.7%, 96.9%]), and 134 of 141 class D (PPA of 95.0% [95% CI, 90.1%, 97.6%]) CPOs (Table 3). The NPA values for class A, class B, and class D CPOs were 99.3% (95% CI, 98.7%, 99.7%), 98.5% (95% CI, 97.7%, 99.1%), and 99.3% (95% CI, 98.7%, 99.7%), respectively.

**TABLE 2:** Ambler classification for clinical and challenge isolates

Group	CPO detect result	No. of isolates that were:					Total
		Class A	Class B	Class D	Indeterminate	CPO negative	
All isolates	Class A	122	0	4	7	4	137
	Class B	3	126	3	1	12	145
	Class D	0	2	134	10	6	152
	Indeterminate	18	27	10	9^*a*^	12	76
	Negative	3	6	0	1	932	942
Total		146	161	151	28	966	1,452

*Enterobacterales* isolates	Class A	106	0	0	7	0	113
	Class B	3	81	1	0	9	94
	Class D	0	2	97	5	1	105
	Indeterminate	15	18	2	1	11	47
	Negative	3	1	0	1	735	740
Total		127	102	100	14	756	1,099

a One of these indeterminate results was for an *Enterobacterales* isolate.

**TABLE 3:** CPO detect test performance, with indeterminate results excluded, for Amber classification, compared to RM results^*a*^

Group	Ambler class	Total no. of tests	No. of CPO detect test results that were:				No. of tests/total, % (95% CI)	
			TP	FP	TN	FN	PPA	NPA
All organisms	A	1,357	122	8	1,221	6	122/128, 95.3 (90.2, 97.8)	1,221/1,229, 99.3 (98.7, 99.7)
	B	1,357	126	18	1,205	8	126/134, 94.0 (88.7, 96.9)	1,205/1,223, 98.5 (97.7, 99.1)
	D	1,357	134	8	1,208	7	134/141, 95.0 (90.1, 97.6)	1,208/1,216, 99.3 (98.7, 99.7)

*Enterobacterales*	A	1,039	106	0	927	6	106/112, 94.6 (88.8, 97.5)	927/927, 100 (99.6, 100.0)
	B	1,039	81	13	942	3	81/84, 96.4 (90.0, 98.8)	942/955, 98.6 (97.7, 99.2)
	D	1,039	97	3	938	1	97/98, 99.0 (94.4, 99.8)	938/941, 99.7 (99.1, 99.9)

a Abbreviations: CPO, carbapenemase-producing organism; TP, true positive; FN, false negative; TN, true negative; FP, false positive; PPA, positive percent agreement; NPA; negative percent agreement; CI, confidence interval.

For *Enterobacterales* alone, CPO detect provided an Ambler classification for 290/343 (84.5%) isolates that were CPO positive by the RM. Thirty-six of 343 (10.5%) of the isolates were Ambler class A, B, or D or indeterminate by the RM but were indeterminate by CPO detect (Table 2). CPO detect correctly classified 106 of 112 class A (PPA of 94.6% [95% CI, 88.8%, 97.5%]), 81 of 84 class B (PPA of 96.4% [95% CI, 90.0%, 98.8%]), and 97 of 98 class D (PPA of 99.0% [95% CI, 94.4%, 99.8%]) RM-positive isolates (Table 3). The NPA values for class A, class B, and class D CPOs were 100% (95% CI, 99.6%, 100.0%), 98.6% (95% CI, 97.7%, 99.2%), and 99.7% (95% CI, 99.1%, 99.9%), respectively.

## DISCUSSION

Given the recent emergence of carbapenem-resistant Gram-negative organisms and their impact on health care around the globe, reliable detection of carbapenem resistance continues to represent a major public health priority (12). While antimicrobial susceptibility testing is generally required to determine the best choice of antibiotic for treatment of infections due to Gram-negative organisms, determination of carbapenemase production and classification of the carbapenemase enzyme may aid therapy decisions (27, 28). In addition, identification of a carbapenem-resistant organism as a CPO has important infection control implications and can inform containment strategies to help prevent the spread of resistance genes (27).

Several methods are used to detect carbapenemase production among carbapenem-resistant *Enterobacterales* and nonfermenting carbapenem-resistant organisms (17, 28). These include phenotypic methods, such as the Carba NP test (29), and the modified carbapenemase inactivation method (mCIM) (30), both of which provide results as carbapenemase positive or carbapenemase negative. A combined disc method that is able to differentiate metallo-β-lactamases (class B) (MBL) from KPC enzymes (class A) has also been reported (31). Matrix-assisted laser desorption-ionization–time of flight (MALDI-TOF) mass spectrometry has been used by some to detect carbapenemase production (32), and a lateral flow immunoassay (Carba5) that is able to detect the five main carbapenemases by type (KPC-, NDM-, VIM-, and IMP-type and OXA-48-like carbapenemases) (33) has also been described. Molecular methods using real-time PCR (34), microarray analysis (35), and whole-genome sequencing (36) have frequently been used to test for and classify carbapenemases. Several of these methods can also be utilized to classify carbapenemase-producing carbapenem-resistant *Enterobacteriaceae* (CP-CRE). These methods of detection differ by target, accuracy of detection, time to result, and level of accessibility (17). While non-phenotype-based assays, such as real-time PCR and microarray analysis, are all associated with relatively high accuracies and same-day turnaround times, they are limited in their ability to target newly resistant strains due to a lack of a nucleotide recognition sequence(s). Phenotypic tests, including multidisc testing, the Carba NP test, the mCIM, and the CPO detect assay, are more likely to identify new CPO strains than those based on molecular methods (28). Due to costs and/or additional time required, many of these tests are performed only if there is suspicion of carbapenemase production (e.g., elevated MICs of carbapenems). In contrast, only CPO detect allows for the simultaneous detection of carbapenemase and Ambler classifications concurrently with routine susceptibility testing.

The results reported here are consistent with those of previous work. The CPO detect test previously showed an effective overall carbapenemase detection capability of >97%, as determined by Thomson et al. (37). Those authors reported findings from a comparison of automated and manual detection methods and classifications of carbapenemases by utilizing CPO detect and the Rapidec Carba NP assay, respectively, on a panel of approximately 300 isolates consisting of 80% *Enterobacterales* and 10% each P. aeruginosa and A. baumannii isolates (37). Croxatto et al. (38) recently reported findings of 97.8% sensitivity and 87.1% specificity for the detection of CPOs by using a retrospective sample set. Although the sensitivity remained high (100%), testing on a prospective sample set resulted in a lower specificity (67.8%) of detection of CPOs (38). Another publication, which included only 190 frozen isolates, reported a slightly lower sensitivity for CPO detection (89.4%) than shown in this study (39). However, the sensitivity suffered in the study by Ong et al. (39), likely due to oversampling of organisms (i.e., Enterococcus cloacae) that express IMI-1, a carbapenemase that is currently not detected with high frequency in most of the world. Although IMI-1 was not included as a target gene in the RM utilized in this study, CPO detect correctly identified all three IMI-containing challenge isolates.

Here, CPO detect returned results for Ambler class A, B, and D, with ≥94.0% accuracy for all organism groups. By Ambler class, Thomson and colleagues (37) reported 85.0%, 72.4%, and 88.6% sensitivities for class A, B, and D organisms, respectively. Importantly, the study by Thomson and colleagues was designed to include high rigor for both assays, as the isolates were chosen to maximize the diagnostic difficulty by including nonroutine isolates (e.g., producers of OXA, KPC-producing A. baumannii, and high-level AmpC producers, in combination with MBL production) that demonstrated diagnostic difficulty with certain carbapenemase testing methods. This may explain why the performance values here were higher than those from the work of Thomson et al. (37). However, Park et al. (40) reported 81.7%, 71.8%, and 82.0% sensitivities for class A, B, and D carbapenemase producers, respectively. Differences between the higher percentages of correct classifications here versus those from Park et al. include values observed for E. coli (98.7% here versus 87.5% from Park et al.), Klebsiella pneumoniae (97.4% here versus 86.6% from Park et al.), P. aeruginosa (95.9% here versus 38.1% from Park et al.), and A. baumannii (95.9% here versus 75.0% from Park et al.) (40).

Identifying carbapenemase production in P. aeruginosa and A. baumannii, in addition to *Enterobacterales*, can be important in some clinical settings (41), but rapid and effective methods of detecting nonfermenting CPOs is not trivial (42). Although nonfermenting Gram-negative bacteria represent a relatively low percentage among CPO-producing organisms (both in the United States and worldwide), carbapenemase activity from these bacteria has been difficult to detect and therefore represents a significant threat. An increasing number of reports have communicated identification of new strains of either A. baumannii or P. aeruginosa in multiple countries, in both public and hospital settings, the latter of which may be especially critical as these organisms are responsible for many nosocomial and opportunistic infections (43). Currently, two phenotypic assays (Carba NP and mCIM) are suggested for use by the CLSI to detect CPO-producing *Enterobacterales* and P. aeruginosa, but not for A. baumannii (16). Additionally, neither of these assays can identify Ambler class, although the mCIM plus the EDTA assay can be used to determine the presence of class B carbapenemase enzymes. Increased access to assays inclusive of A. baumannii are critical if a comprehensive approach toward suppressing carbapenem resistance is to be achieved in the United States and abroad.

There are some limitations of the findings presented here. The study RM included multiplex PCR assays that targeted the most common carbapenemase genes related to Ambler class A (KPC family), class B (IMP, NDM, and VIM families), and class D (OXA-48 and OXA-48-like, OXA-23 and OXA-23-like, OXA-24 and OXA-24-like, and OXA-58 and OXA-58-like genes), and classification performance was not fully evaluated for other carbapenemase genes, including *bla*_GES_ (*n* = 3), *bla*_IMI_ (*n* = 3), *bla*_SME_ (*n* = 12), and *bla*_SPM_ (*n* = 1), which were identified by CPO detect. In addition, isolates that contain more than one carbapenemase gene, specifically genes of multiple Ambler classes, may result in a CPO-positive test result with an Ambler classification that is either indeterminate (i.e., Amber classification not provided) or class D. This occurred with seven isolates (which were excluded from classification analysis in this article) during the conduct of this study. Due to this small group number, no conclusion regarding the classification performance of CPO detect with organisms coproducing multiple classes of carbapenemases can be made. The specificity of CPO detect when testing carbapenem-resistant *Enterobacterales*, P. aeruginosa, or A. baumannii isolates known to express a cephalosporinase or an ESBL—with either enzyme coupled with porin mutations—has not been fully evaluated. Out of 734 carbapenem-nonsusceptible isolates in the clinical study, only four were well-characterized strains with such a known resistance mechanism. All four strains were falsely reported as carbapenemase producers by CPO detect. As with other phenotype-based tests, some studies have shown that the specificity of detection of CPO detect depends on the phenotype of the non-CPOs; decreased specificity is expected with testing of challenging isolates that, for example, express ESBLs and/or AmpCs in combination with porin or efflux mutations (37). Previous studies have reported a higher percentage of false positives than shown here (37, 38). This may be due, at least in part, to the inclusion of challenge sets composed of a high percentage of isolates that express ESBL or AmpCs in the presence of mutations, such as porin or efflux channels. Consistently with this possibility, CPO detect returned 14 false-positive results from the total 82 negative reference results (NPA = 82.9%) within the challenge set, whereas it returned only 20 false positives within the total 884 reference-negative results (NPA = 98.9%) from a more routine clinical set. The lower NPA result from the challenge set here is similar to the trend for NPA determined for all non-CPO, carbapenem-resistant organisms by both CPO detect (68.6%) and Rapidec Carba NP (78.4%, with borderline results interpreted as negative) by Thomson and colleagues (37).

Finally, the algorithm comprising the composite reference method utilized multiple testing methods to ensure an accurate CPO status for both fermenting and nonfermenting Gram-negative organisms targeted in this study. The utilization of mCIM for the detection of A. baumannii was shown previously (44) to be suboptimal (using CLSI criteria). However, as shown in Fig. 2, both positive and negative mCIM results were supported by supplemental testing in order to insulate potential false-positive and -negative A. baumannii (or *Enterobacterales* or P. aeruginosa) results. In addition, internal validation of the mCIM method with A. baumannii produced a sensitivity and a specificity of 87.2% and 100%, respectively, for carbapenemase-positive organisms (data not shown).

Many of the latest approved drugs, including ceftazidime-avibactam and meropenem-vaborbactam, are not highly active against Ambler class B carbapenemases, whereas they may be active against other carbapenem classes, especially class A. Thus, misclassification of class B as a false positive or a false negative may result in suboptimal patient care, in which the patient recovers more slowly from infection or does not recover at all. Misclassification and subsequent mistreatment may also contribute to an increase in organism resistance over time. Here, the percentages of CPO detect Ambler class A results that were either class B, class D, or indeterminate by the reference method were 0% (0/133), 3.0% (4/133), and 5.3% (7/133), respectively. The percentages of CPO detect Ambler class B results that were either class A, class D, or indeterminate by the reference method were 2.3% (3/133), 2.3% (3/133), and 0.8% (1/133), respectively (Table 2).

### Conclusions.

The high mortality and continuing emergence of resistance associated with infections caused by CPO suggest that there is an increasing need for laboratories to have the ability to provide rapid and accurate CPO detection (45). As in previous studies (37, 38), this study showed a high sensitivity of detection. The specificity of CPO detect may be impacted by the percentage of carbapenem-resistant isolates tested. It is important from a clinical standpoint to determine the conditions that would be required to warrant additional testing for the presence of carbapenemase following a positive result by any phenotype-based CPO test. However, simultaneous susceptibility testing, CPO detection, and Ambler class identification may benefit patient health care by reducing the need for additional testing, providing information for clinicians during diagnoses and subsequent patient treatment and helping to mitigate the spread of resistance genes (27).

Becton, Dickinson and Company, BD Life Sciences-Diagnostic Solutions’ 510(k) premarket notification filing (K181665) can be found at https://www.accessdata.fda.gov/scripts/cdrh/cfdocs/cfpmn/pmn.cfm.

## Supplementary Material

Supplemental file 1
