# Supplementary material for: Multicenter Evaluation of the BD Phoenix CPO Detect Test for Detection and Classification of Carbapenemase-Producing Organisms in Clinical Isolates
Source: J Clin Microbiol. 2020 Apr 23;58(5):e01752-19. doi: 10.1128/JCM.01752-19 (PMC7180248; doi:10.1128/JCM.01752-19)
Supplement: Supplemental file 1 [file JCM.01752-19-s0001.pdf]

Table S1. Summary of *in silico* results for variants included in the PCR multiplex method.

**Target variants found in Genbank based on *in silico* analysis**[a]
65 IMP's: IMP1-35, 36-38; 40-56, 58-64, 66-69
26 KPC's: KPC 1-20, 27-28,30-32
20 NDM's: NDM 1-19; NDM 21
12 OXA-48 & like: 48; 162, 163, 181, 199, 204,232, 244, 245, 247, 370 and 484
53 VIM's: VIM 1-52; 54
23 OXA-23 & like: 23; 27; 49; 103; 146; 165-171; 225; 239; 366;398; 422-423; 435; 440;482;565
9 OXA-24 & like: 24-26; 40; 72; 139;160; 207, 437
7 OXA-58 & like:58; 96-97; 164; 397; 420;512

[a]*In silico* analysis was performed to confirm the PCR primers in the multiplex assays properly align with known gene target variants. This study was based on extracting published sequences of CPO target variants from GenBank® and aligning the variants for confirmation of hybridization using the software tool Geneious R11. The *in silico* study showed that these variants tested by the PCR multiplex method correctly aligned along the proper amplified gene variant type and fragment size with the primer sequences.

Table S2. CPO detect Test Performance for carbapenemase detection within *Enterobacterales* organisms compared to RM

Species	Total	RM Neg	RM Pos	FP	FN	% Agreement
Citrobacter freundii	28	17	11	0	0	100
Citrobacter koseri	19	18	1	0	0	100
Citrobacter species	4	4	0	0	0	100
Enterobacter cloacae	79	46	33	0	3	96.2
Enterobacter species	1	1	0	0	0	100
Escherichia coli	238	183	55	3	0	98.7
Klebsiella aerogenes	33	29	4	1	0	97.0
Klebsiella oxytoca	36	31	5	1	1	94.4
Klebsiella pneumoniae	418	231	187	11	0	97.4
Klebsiella species	1	0	1	0	0	100
Miscellaneous enteric[a]	3	2	1	0	0	100
Morganella morganii	29	27	2	0	0	100
Pantoea agglomerans	1	1	0	0	0	100
Proteus mirabilis	42	36	6	2	1	92.9
Proteus vulgaris/penneri	23	23	0	1	0	95.7
Providencia species	26	21	5	1	0	96.2
Providencia stuartii	18	16	2	1	0	94.4
Salmonella species	18	17	1	0	0	100
Salmonella typhi	2	2	0	0	0	100
Serratia marcescens	57	33	24	0	0	100
Serratia species	7	2	5	0	0	100
Shigella sonnei	9	9	0	0	0	100
Shigella species	7	7	0	0	0	100

Abbreviations: CPO, carbapenemase producing organism; RM, reference method; Neg, negative; Pos, positive; FP, false positive; FN, false negative

[a]Miscellaneous enteric organisms included *Kluyvera ascorbate* (n=2) and *Leclercia adecarboxylata* (n=1)
